# Gentamicin and clindamycin antibiotic-eluting depot technology eradicates *S. aureus* in an implant-associated osteomyelitis pig model without systemic antibiotics

**DOI:** 10.1128/aac.00691-24

**Published:** 2024-09-17

**Authors:** Nicole L. Henriksen, Elizabeth Serrano-Chávez, Albert Fuglsang-Madsen, Louise K. Jensen, Hans Gottlieb, Mats Bue, Thomas L. Andresen, Jonas R. Henriksen, Anders E. Hansen

**Affiliations:** 1 Department of Health Technology, Technical University of Denmark, Kgs. Lyngby, Denmark; 2 Department of Veterinary and Animal Sciences, University of Copenhagen, Frederiksberg, Denmark; 3 Department of Orthopedic Surgery, Herlev Hospital, Herlev, Denmark; 4 Department of Clinical Medicine, Aarhus University, Aarhus, Denmark; 5 Department of Orthopaedic Surgery, Aarhus University Hospital, Aarhus, Denmark; Columbia University Irving Medical Center, New York, New York, USA

**Keywords:** antibiotics, osteomyelitis, orthopedic, drug delivery, drug-eluting depot

## Abstract

The therapeutic challenges of orthopedic device-related infections and emerging antimicrobial resistance have attracted attention to drug delivery technologies. This study evaluates the preclinical efficacy of local single- and dual-antibiotic therapy against implant-associated osteomyelitis (IAO) using a drug-eluting depot technology, CarboCell, that provides sustained release of high-dose antibiotics and allows for strategic *in situ* placement in relation to infectious lesions. Clindamycin and gentamicin were formulated in CarboCell compositions. One-stage-revision of tibial *Staphylococcus aureus* IAO was conducted in 19 pigs. Pigs were treated locally with CarboCell containing either gentamicin alone for 1 week or a co-formulation of gentamicin and clindamycin for 1 or 3 weeks. Bone, soft tissue, and antibiotic depots were collected for microbiology, histology, and HPLC analyses. Supporting *in vivo* release studies of CarboCell formulations were performed on mice. Both single- and dual-antibiotic CarboCell formulations were developed and capable of eradicating the infectious bacteria in bone and preventing colonization of implants inserted at revision. Eradication in soft tissue was observed in all pigs after 3 weeks and in 6/9 pigs after 1 week of treatment. Neutrophil counts in bone tissue were below the infection cut-off in all pigs receiving the dual-antibiotic therapies, but above in all pigs receiving the single-antibiotic therapy. Histological signs of active bone reorganization and healing were observed at 3 weeks. In conclusion, all CarboCell formulations demonstrated strong therapeutic activity against IAO, eradicating *S. aureus* in bone tissue and preventing colonization of implants even without the addition of systemic antibiotic therapy.

## INTRODUCTION

Orthopedic device-related infection (ODRI) represents a tremendous clinical challenge that is associated with high treatment failure rates and substantial patient and socioeconomic burdens. Development of effective preventive and therapeutic strategies is therefore a critical objective in the field of orthopedic medicine (1). ODRIs are challenging as biofilm formation on implants and within bone tissue promotes tolerance toward the host immune system and antimicrobial treatment (2). The rise in antimicrobial resistance (AMR) has attracted more attention to combination-based treatments and the development of effective drug delivery technologies (3, 4). Antibiotic-eluting technologies have thus become widely used in the treatment of ODRI as they aim to provide high, sustained, local concentrations of antibiotics in bone tissue, which can be difficult to achieve by systemic administration, while minimizing the side effects of long-term systemic therapy (5, 6). However, since antibiotic-eluting bone cement was first introduced in the 1970s (7), only few technologies have become commercially available (5). Novel technologies have the potential to address the concerns associated with the use of bone cement, such as lack of standardized formulation protocols and prolonged release of subtherapeutic concentrations of antibiotics, which potentially can select for AMR (8).

Gentamicin is a commonly used antibiotic in local treatment of ODRI due to its broad antibacterial and concentration-dependent activities (5, 9). The hydrophilic nature of antibiotics like gentamicin can, however, hinder the efficient formulation and sustained release from hydrophobic vehicle systems, with potential implications for clinical outcomes (10). Chemical modification using hydrophobic ion-pairing (HIP), in which hydrophilic compounds are coordinated to amphiphilic counterions to form hydrophobic salts, may help overcome these issues and increase the performance of antibiotics in drug delivery technologies (10, 11). While gentamicin is commonly used as a single local therapy, *in vitro* studies suggest that gentamicin and clindamycin released from bone cement display antibacterial synergism against common ODRI pathogens like *Staphylococcus aureus* (9, 12), including gentamicin-resistant staphylococcal strains (13). However, despite *in vitro* evidence in favor of local combination-based therapy, few *in vivo* data exist to support this for the treatment of established ODRI (14).

**Fig 1:**
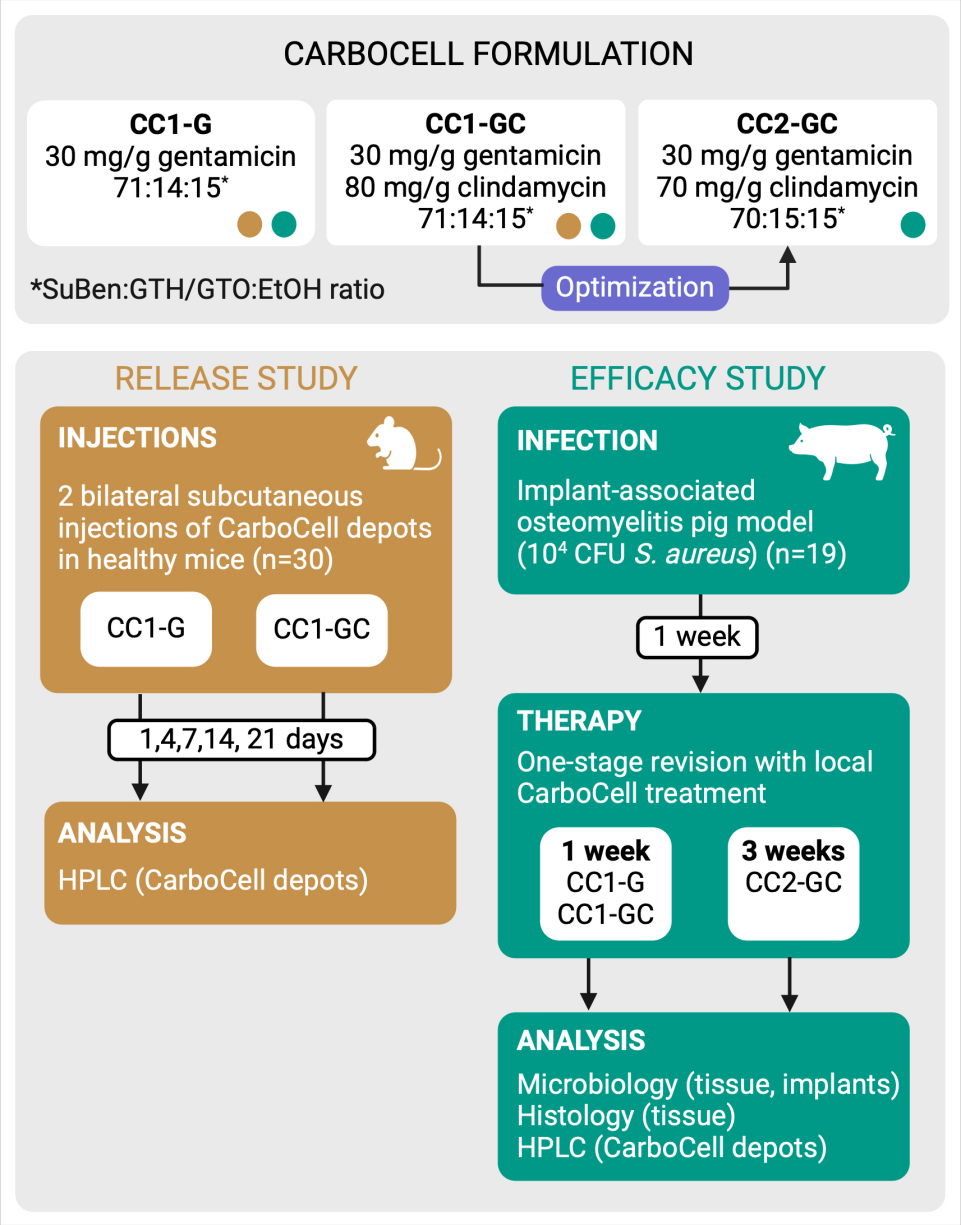
Study overview. CarboCell formulations containing either gentamicin (CC1-G) or a combination of gentamicin and clindamycin (CC1-GC) were prepared using hydrophobic ion-pairing of gentamicin and docusate. *In vivo* release of both antibiotics from subcutaneous CC1-G and CC1-GC depots was assessed in mice over 1–21 days. The CarboCell formulation was improved to provide better injectability and translatability, generating CC2-GC. The efficacy of local treatment with all three CarboCell formulations was tested in a pig model of one-stage revision of implant-associated osteomyelitis over 1 or 3 weeks.

Therefore, the purpose of this study was to evaluate the efficacy of HIP-modified gentamicin alone or in combination with clindamycin against *S. aureus* implant-associated osteomyelitis (IAO) in a pig model. The antibiotics were delivered locally using the drug-eluting depot technology, CarboCell, without systemic antibiotics (Fig. 1). CarboCell is a ready-to-use injectable carbohydrate ester-based liquid formulation developed to facilitate strategic placement of drug depots in relation to infectious foci and provide sustained release of antibiotics for prevention and treatment of bacterial infections. In initial preclinical studies, CarboCell showed sustained release of clindamycin, resulting in favorable prophylactic and therapeutic outcomes for IAO (15). Using HIP, we now expand the drug compatibility of CarboCell to encompass charged hydrophilic antimicrobials like gentamicin and antibiotic combinations.

## MATERIALS AND METHODS

### CarboCell formulation and HIP

#### Ion-pairing of gentamicin

The hydrophobic ion-pairing of gentamicin and docusate was prepared by dissolving 8 g gentamicin in 160 mL 0.01M HCl and dissolving 19.2 g sodium docusate in 320 mL MeOH. The single-phase mixture was shaken in a separation funnel for 5 minutes, whereafter 160 mL MilliQ water and 160 mL chloroform were added, resulting in a biphasic solution. The organic phase was collected before gentamicin–docusate was obtained by rotary-evaporation.

#### Desalting of clindamycin

Desalted clindamycin was prepared by extracting clindamycin hydrochloride monohydrate. Briefly, 10 g clindamycin hydrochloride monohydrate was dissolved in 100 mL MilliQ water and mixed with 200 mL chloroform in a separation funnel. NaOH (~10 mL, 2 mM) was added in sequential steps and shaken until the pH of the aqueous top phase reached ~11. The organic phase was collected, and desalted clindamycin was obtained by rotary-evaporation.

#### Preparation of CarboCell formulations

CarboCell compositions were prepared by weighing the carbohydrate ester sucrose benzoate (SuBen), triglyceride glycerotrihexanoate (GTH) or glycerotrioctanoate (GTO), and ethanol (EtOH) into a sealed glass vial in the ratios SuBen:GTH:EtOH (71:14:15% wt/wt, formulation CC1) and SuBen:GTO:EtOH (70:15:15% wt/wt, formulation CC2). The mixture was heated at 60°C and sonicated for 30–60 minutes until homogeneous.

Three CarboCell formulations were prepared by solubilizing specified amounts of either desalted clindamycin or gentamicin–docusate in these. For the CC1-G formulation, 30 mg gentamicin (111 mg gentamicin–docusate) was dissolved per gram SuBen:GTH:EtOH (71:14:15% wt/wt) CarboCell solution. For the CC1-GC formulation, 30 mg gentamicin (111 mg gentamicin–docusate) and 80 mg desalted clindamycin were dissolved per gram SuBen:GTH:EtOH (71:14:15% wt/wt) CarboCell solution. For the CC2-GC formulation, 30 mg gentamicin (111 mg gentamicin–docusate) and 70 mg desalted clindamycin were dissolved per gram SuBen:GTO:EtOH (70:15:15% wt/wt) CarboCell solution. Compared to CC1, the CC2 composition had reduced viscosity and improved quality of the triglyceride component. The rationale for these improvements is outlined in the *Results* section.

### Experimental animal work

#### *In vivo* release in mice

Thirty 8–9-week-old, female, Balb/C mice (Taconic Biosciences, USA) were randomized to receive either CC1-G or CC1-GC. Mice were anesthetized with isoflurane and received bilateral 50-µL subcutaneous injections of CarboCell on the back. Three randomly selected mice from each group were euthanized by cervical dislocation at 1, 4, 7, 14, and 21 days post-injection, and depots were collected for high-performance liquid chromatography (HPLC) analysis.

#### One-stage revision in an IAO pig model

A well-characterized model of IAO was established in 10 pigs (Danish Landrace, specific-pathogen-free, female, 2–3 months old, average weight: 32 kg). Briefly, a 4 × 20 mm hole was drilled in the right proximal tibial bone and inoculated with 10^4^ CFU/10 µL *S*. *aureus* S54F9 (biofilm-producing strain isolated from a porcine lung abscess), followed by insertion of a 2 × 15 mm steel implant (16, 17). A surgical one-stage revision procedure was performed after 1 week, comprising debridement and sampling of infected and necrotic soft- and bone tissue, wound and bone-void irrigation, and implant exchange, as previously described (18). The pigs were randomized to receive local treatment with either CC1-G or CC1-GC. CarboCell was injected into the bone void and adjacent vital trabecular bone- and soft tissue (average injected volume: 8.1 mL (range: 7–10.5 mL)). Post-operative analgesia was managed daily with oral meloxicam (Metacam, Boehringer Ingelheim, Denmark) and supplemented with intramuscular injections of buprenorphine (Bupaq, Salfarm, Denmark) upon indication. The pigs were euthanized with pentobarbital (Euthanimal, ScanVet, Denmark) 1 week after CarboCell treatment. Necropsy and macroscopic assessment of the surgical wound and tibial bone lesion were performed in all pigs. To assess the prolonged effects of treatment and dose reduction, a follow-up experiment was conducted in which 12 pigs were treated with CC2-GC for 3 weeks, reducing the amount of injected CarboCell by approximately one-third (average injected volume: 5.8 mL (range: 4–7.5 mL)). The IAO pig model has been extensively characterized and demonstrated as not able to spontaneously clear the *S. aureus* infection (19–21). We therefore refrained from including further untreated controls but assessed that all animals had established infections before debridement and treatment.

During the study periods, three pigs were euthanized due to lameness before or shortly after revision surgery (related to the IAO infection or hematoma at the surgical site). One pig (CC1-G group) euthanized on day 12 due to wound dehiscence was included in statistical analyses, and final group sizes were therefore CC1-G (*n* = 4), CC1-GC (*n* = 5), and CC2-GC (*n* = 10).

### Histopathology

Formalin-fixed, decalcified and paraffin-embedded sections of bone containing the lesion were cut into 4- to 5-μm-thick sections. Tissue sections were stained with H&E, Masson’s trichrome for collagen, and an immunohistochemical stain for detection of *S. aureus* (22, 23) and assessed similarly to previous studies (21). Briefly, within the pathological bone area (PBA) (17), the following parameters were assessed: (i) neutrophil count: neutrophils were quantified in 10 high-power fields (HPF, x40) using the European Bone and Joint Infection Society’s histological definition of infection of ≥five neutrophils in ≥five HPFs (24); (ii) osteoclast count: a maximum of 10 osteoclasts in 10 HPFs were counted (20); (iii) fibrosis score: the percentage of collagen in 10 fields (x20) was scored on a semi-quantitative scale (0: <5%, 1: 5%–10%, 2: 10%–25%, 3: 25%–50%, 4: 50%–75%, 5: 75%–100%) and averaged (25). Additionally, the presence of *S. aureus* and pathomorphological response to CarboCell were determined in the whole tissue section. Finally, paraffin-embedded sections of renal tissue were stained with H&E to assess gentamicin-related toxicity. All histological evaluations were conducted blinded by a single observer.

### Microbiology and modified disk diffusion assay

Microbiological analyses were conducted as previously described (15). Briefly, 3–5 bone tissue and three soft tissue samples from each pig were collected at revision and post-mortem. Samples were homogenized (Retsch MM 400, 1,500 rpm, room temperature, 10 minutes), centrifuged (5,000 x g, 4°C, 10 minutes), and the pellet resuspended in 1 mL PBS. Tissue samples and implants, collected in PBS, were sonicated (Branson 1510 sonicator, 4°C, 10 minutes) and serially diluted before 30 µL of each sample was spot-plated and incubated at 37°C overnight (detection limit: 33.33 CFU/mL). The colony-forming units (CFUs) per g tissue or cm^2^ implant were calculated for each sample and averaged for each pig. The presence of *S. aureus* was confirmed by an agglutination test (Staphaurex Latex Agglutination Test, Thermo Fisher Scientific, USA) or by mannitol salt agar culture. In cases of sterile samples, the entire volume was plated out to confirm the eradication of *S. aureus*. Other isolates were identified using matrix-assisted laser desorption ionization-time of flight mass spectrometry (MALDI-TOF, Vitek MS RUO, bioMérieux, France) (26).

To assess the development of antibiotic resistance in surviving bacteria, a modified disk diffusion assay was performed as previously described (15). Briefly, 100 µL of a diluted overnight culture of *S. aureus* (strain S54F9) was spread across a 1.5% wt/wt lysogeny broth agar plate. Holes were made in the agar, and 25 µL of Milli-Q water and 50 µL of 4 mg/mL clindamycin (0.2 mg) or 2 mg/mL gentamicin (0.1 mg) were added to each hole. Plates were incubated at 37°C overnight, and clearing zones were measured.

### HPLC

CarboCell depots collected from *in vivo* studies were analyzed for gentamicin and clindamycin concentrations on a Nexera-i HPLC system (Shimadzu, Kyoto, Japan) with an evaporative light scattering detector (ELSD). Each CarboCell depot was dissolved in DMSO at ~50 mg/mL. Samples were filtered using 0.45-µm pore nylon syringe filters and diluted up to five times with DMSO. Samples (5 µL) were injected onto a Waters HSS-T3 column at a flow rate of 0.5 mL/min. The solvent system consisted of mobile phase A (5% MeCN, 20 mM HFBA in water) and mobile phase B (20 mM HFBA in MeCN). The gradient was 0% B for 1.5 minutes, 0 to 100% B over 8 minutes, 100% B for 2 minutes, 100% B to 0% B over 0.5 minutes, and 0% B for 2 minutes. Gentamicin and clindamycin were detected by ELSD and SuBen by UV–vis absorption (280 nm). Standards in triplicates of 40, 100, 200, 400, 500, 600, and 750 µg/mL clindamycin or gentamicin and 0.3, 0.6, 1.2, 1.8, 3.6, 5.4, 7.5, and 15 mg/mL SuBen were prepared, measured, and quantified in GraphPad (Version 10.0.3). Clindamycin and gentamicin were analyzed by nonlinear (y = aC^b^) 1/SD^2^ weighed regression, whereas SuBen was analyzed by linear 1/SD^2^ weighed regression. Obtained goodness of fits were R^2^ >0.998 for all calibration plots, LOQ (gentamicin) =16 µg/mL, LOQ (clindamicin) =31 µg/mL and LOQ (SuBen) =0.1 mg/mL (27, 28). Accuracy and precession were gaged by %RSD <7% and %Recovery 98%–105% for gentamicin in the range 100–750 µg/mL, %RSD <6% and % Recovery 99-102% for clindamycin in the range 100–750 µg/mL, and %RSD <3% and %Recovery 96%–102% for SuBen in the range 0.3–15 mg/mL. The drug release was reported relative to the input, i.e., the initial CarboCell antibiotic to SuBen concentration ratio (C_AB_/C_SuBen_)^initial^. The release was thus calculated as %Rel = 1-(C_AB_/C_SuBen_)/(C_AB_/C_SuBen_)^initial^ for every timepoint, where C_AB_ and C_SuBen_ are the antibiotic and SuBen concentration of the dissolved CarboCell depot, respectively.

### Cell viability assay

A cell viability assay was conducted to investigate the influence of gentamicin and docusate on osteoblasts. An immortalized murine myoblast cell line, C2C12, transdifferentiated into osteoblasts, was used (29). Cells were cultured in DMEM high glucose medium supplemented with 10% FBS and penicillin/streptomycin at 37°C and 5% CO_2_ until confluent. Cells were seeded in a 96-well plate (1 × 10^4^ cells/well), and incubated for 24 hours. Gentamicin and docusate were dissolved in Milli-Q water; added to wells at concentrations of 0.1, 1, 10, 100, 250, 500, and 1,000 and incubated for 24 hours. A no drug control and media blank control were included, with everything in triplicates. The CyQUANT^TM^ MTT cell viability assay (Thermo Fisher Scientific) was conducted according to the manufacturer’s instructions. Briefly, the medium was replaced, and 10 µL MTT reagent (12 mM) was added and incubated for 18 hours. Then, 100 µL of SDS-HCl was added and incubated for 4 hours. The absorbance was measured on a Spark multimode microplate reader (Tecan, Männedorf, Switzerland) at 570 nm. The background signal from the media blank was subtracted, and cell viability was calculated as a percentage of the negative control.

### Statistics

Statistical analyses were conducted in R (Version 4.3.2, R Foundation for Statistical Computing). Data were analyzed using two-sample *t*-tests (*in vivo* release) and one-way ANOVA (histology, cell viability, and revision microbiology). Assumptions of homogeneity of variance and normal distribution of residuals were assessed, and data were transformed when necessary. +1 was added to all microbiology data, which were then log-transformed. Nonparametric data were analyzed by a Mann–Whitney or Kruskal–Wallis test. Histology and cell viability data were corrected for multiple comparisons using Tukey’s or Dunn’s and Dunnett’s tests, respectively. Data are presented as means with standard deviations (SD) or percentages, and *P*-values below 0.05 were considered statistically significant. Figures were created with GraphPad Prism (version 10.0.3) and www.biorender.com.

## RESULTS

### Gentamicin–docusate and desalted clindamycin can be formulated in CarboCell

The sulfonic acid, docusate, resulted in effective formation of gentamicin–docusate ion-pairs, as previously reported (10). Gentamicin–docusate was found to have high solubility of up to 50 mg gentamicin per gram CarboCell, which was insoluble in its native salt form. Subsequent HPLC analysis of the isolated gentamicin–docusate ion-pair revealed % wt/wt (gentamicin/gentamicin–docusate) of 27%, corresponding to a stoichiometry of 1:3 for gentamicin to docusate. Desalting clindamycin hydrocholoride by extraction enhanced the solubility up to 80 mg clindamycin per gram CarboCell.

The *in vivo* release of gentamicin was initially assessed by subcutaneous injection in mice using a previously reported CarboCell composition (SuBen:GTH:EtOH (60:20:20% wt/wt)) (15). This resulted in complete drug release after approximately 9–10 days, with an initial 52% release after 24 hours (Fig. S1A). Subsequently, the initial release was fine-tuned, reducing it to 29±2% through successive adjustments of GTH and EtOH contents (Fig. S1B), resulting in the composition SuBen:GTH:EtOH (71:14:15% wt/wt), which was used for CC1-G and CC1-GC formulations. To further translation, a third dual antibiotic CarboCell (CC2-CG) with the composition SuBen:GTO:EtOH (70:15:15% wt/wt) was developed. CC2-GC had enhanced injectability due to its reduced viscosity (Fig. S1C) and is based on the triglyceride GTO, which is available in GMP quality.

### Sustained release is achieved for single and dual antibiotic CarboCell formulations

**Fig 2:**
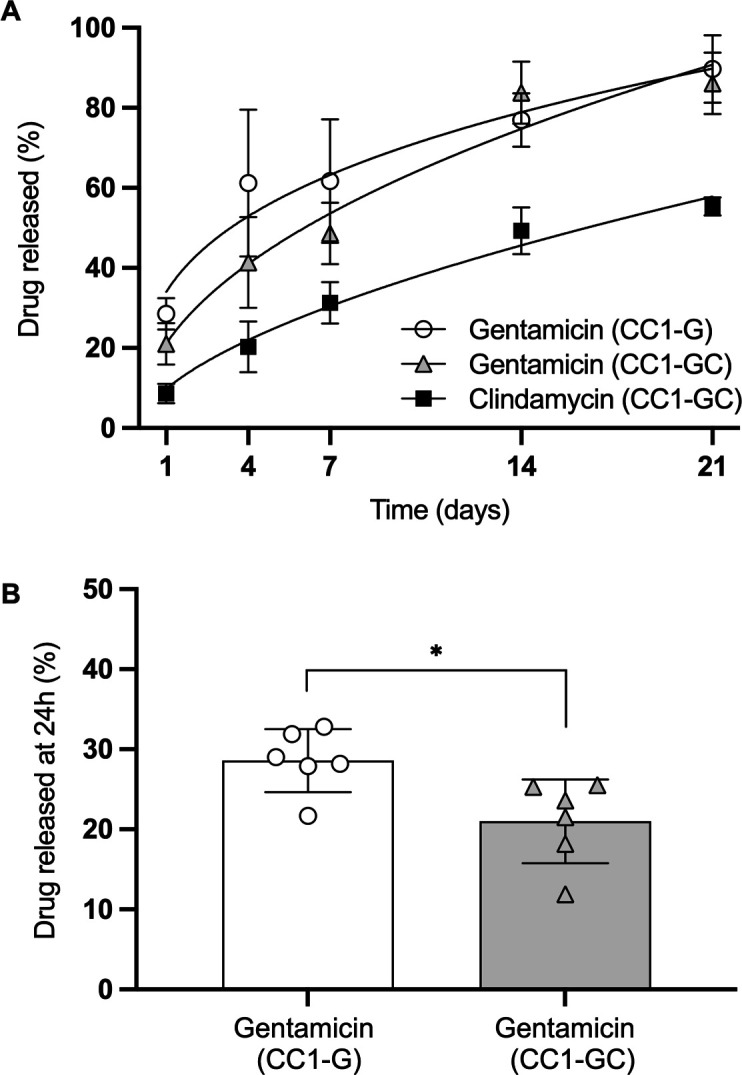
Mice: antibiotic release from CarboCell depots in subcutaneous tissue. (**A**) Accumulated release of gentamicin and clindamycin from subcutaneous CC1-G and CC1-GC depots in mice over 21 days. (**B**) 24-h time point from (**A**) depicting differences in the initial release of gentamicin between formulations. The asterisk denotes statistical probability levels below 0.05.

**Fig 3:**
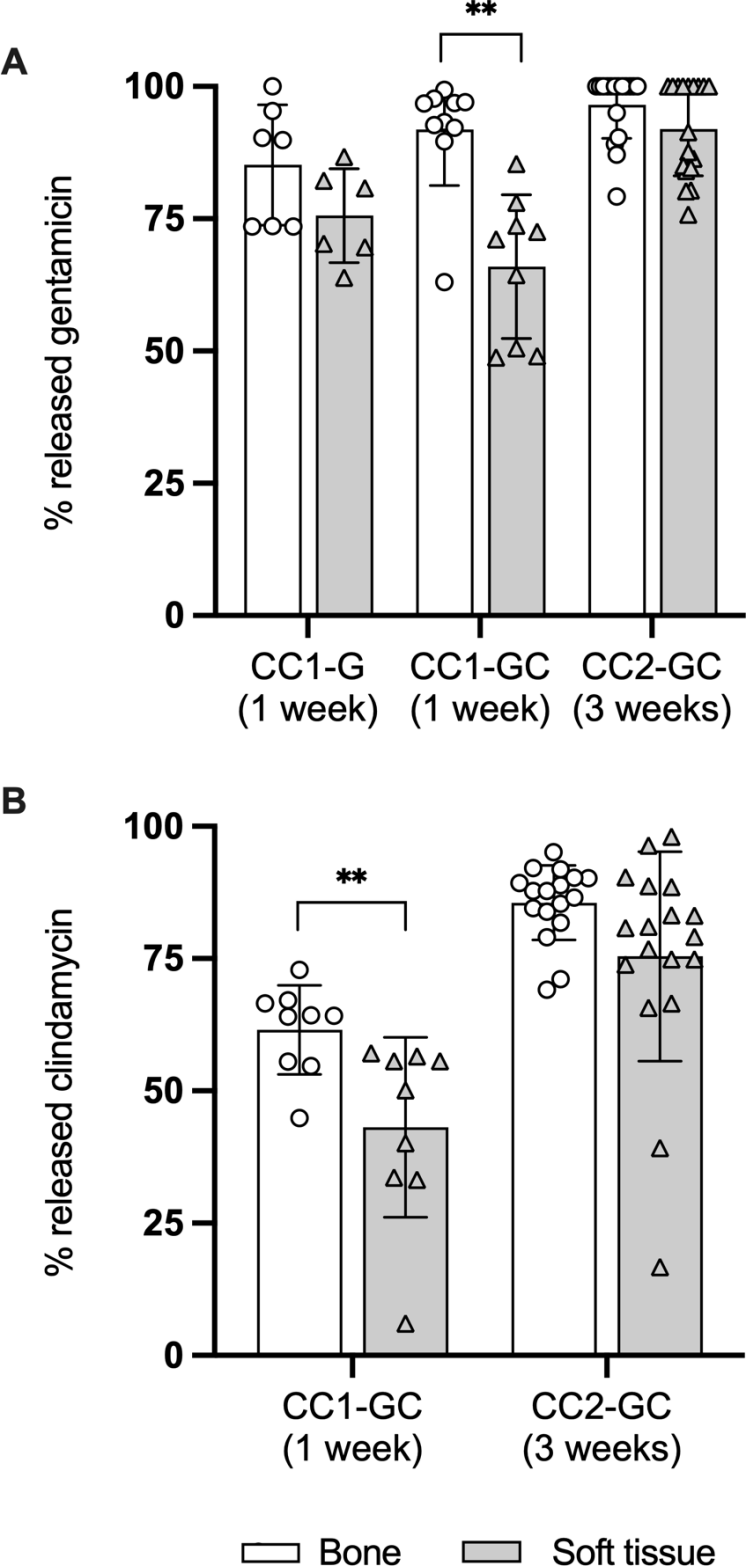
Pigs: antibiotic release from CarboCell depots in bone and soft tissue. Accumulated release of gentamicin (**A**) and clindamycin (**B**) from CarboCell depots in bone and soft tissue from pigs treated with CC1-G, CC1-GC, or CC2-GC for 1–3 weeks. Double asterisks denote a statistical probability level below 0.01.

CarboCell formulations displayed sustained release of incorporated antibiotics. *In vivo* release of gentamicin from CC1-G and CC1-GC depots in subcutaneous tissue in mice reached 90±8% and 86±8 %, respectively, over 21 days, whereas clindamycin was released from CC1-GC depots at a slower rate reaching 55±2% (Fig. 2A). The addition of clindamycin reduced the initial 24-hour release of gentamicin from 29±4% in the CC1-G group to 21±5% in the CC1-GC group (*P* < 0.05) (Fig. 2B). The recovered depots from pigs also displayed sustained release of antibiotics with a slightly faster release of gentamicin relative to clindamycin and with depots in bone initially (after 1 week) displaying a faster release relative to depots recovered from soft tissue (CC1-GC: *P* < 0.01) (Fig. 3A and B). Generally, there was a very homogenous release between depots. The few depots that display low release could be related to unintentional sampling and analysis of parts of a larger depot (Fig. 3A and B).

### CarboCell eradicates *S. aureus* in bone tissue and prevents implant colonization

**Fig 4:**
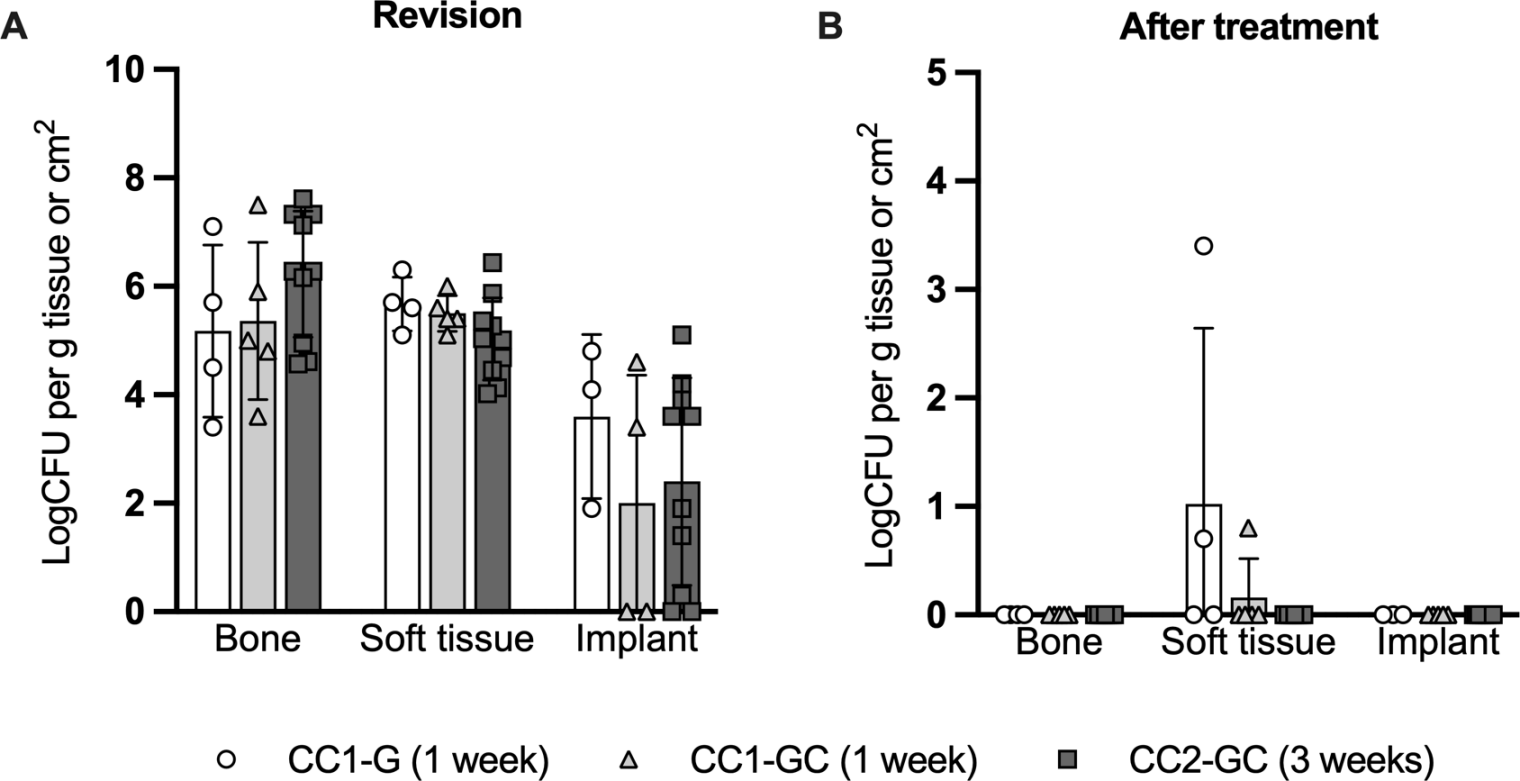
Microbiological efficacy of CarboCell treatment. Bacterial load (log CFU/g or log CFU/cm^2^) of *S. aureus* in bone, soft tissue, and on implants at revision (**A**) and following 1–3 weeks of CC1-G, CC1-GC, or CC2-GC treatment in pigs (**B**). Implants were not recovered in 2 CC1-G, 1 CC1-GC, and 2 CC2-GC pigs.

Bone- and soft tissue samples from all pigs were infected at revision, with no statistical difference between treatment groups (bone: *P* = 0.3, soft tissue: *P* = 0.2). Four implants were sterile, but there were likewise no differences between groups (*P* = 0.5) (Fig. 4A). Following 1 week of treatment with CC1-G or CC1-GC and 3 weeks of treatment with CC2-GC, *S. aureus* was not detected in any bone tissue samples or on implants (Fig. 4B). Two CC1-G and one CC1-GC pigs had *S. aureus-*infected soft tissue samples but displayed 1.7–4.9-fold reductions in log CFU/g tissue (Fig. 4B). None of the infecting isolates developed resistance toward gentamicin or clindamycin, albeit some had reduced sensitivity (Table S1). Bacterial contamination was detected in soft tissue samples from seven pigs, one implant and bone samples from four pigs, with *Pseudomonas fragi* and *Staphylococcus epidermidis* being the dominant species. This contamination is believed to originate from necropsy/storage and does not affect the ability to detect *S. aureus*.

### The bone pathomorphological response to CarboCell treatment

**Fig 5:**
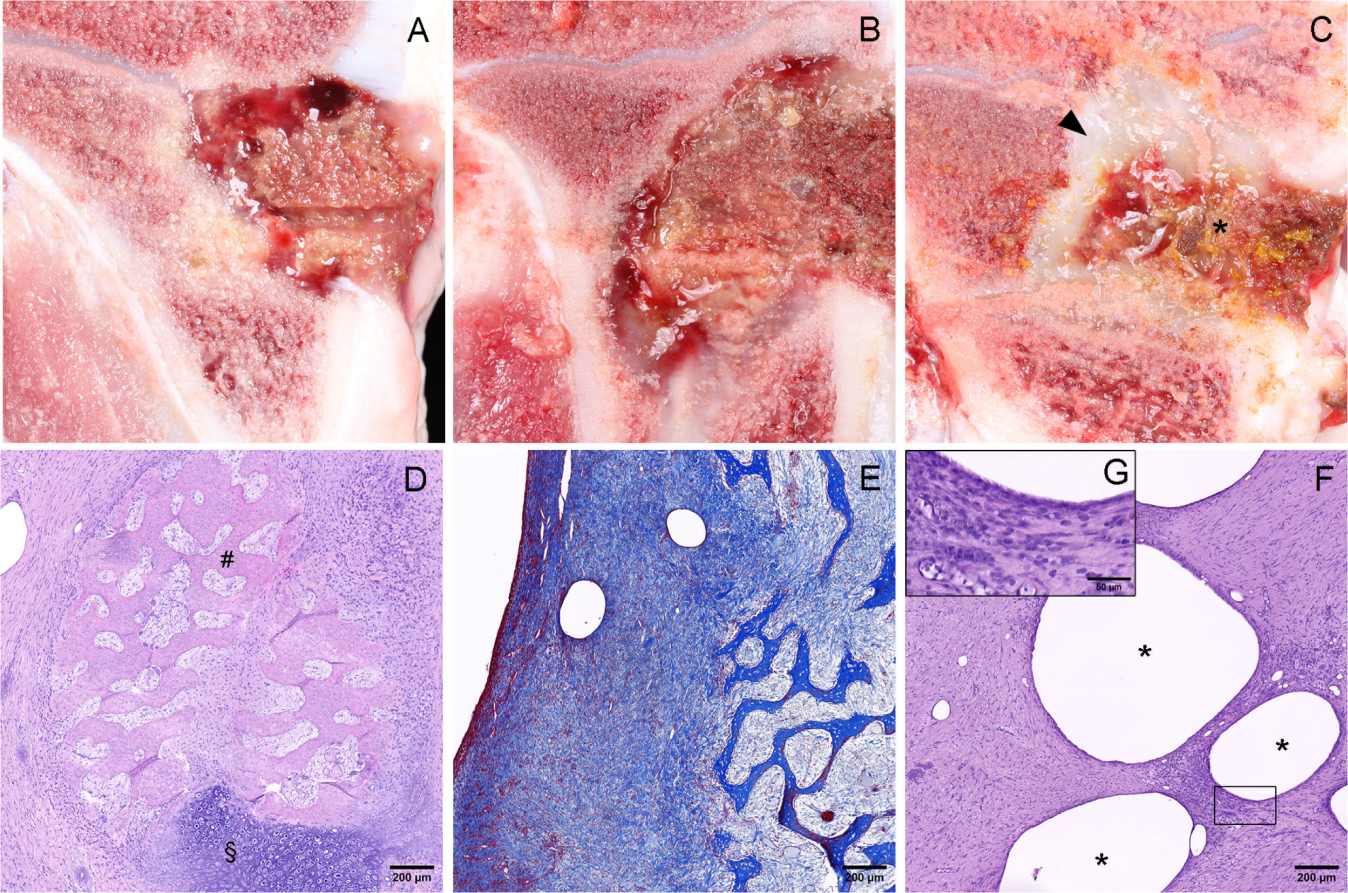
Pathological and histopathological findings. (**A–C**) Macroscopic appearance of bone tissue in CC1-G- (**A**), CC1-GC- (**B**) and CC2-GC-treated pigs (**C**), depicting the interaction between CarboCell and the organizing hematoma (*), and fibrous tissue formation (arrowhead). (**D**) H&E-stained bone tissue section showing a foci of newly formed cartilage (§) and woven bone (#) after 3 weeks of treatment with CC2-GC. (**E**) Masson trichrome-stained bone tissue section from a CC2-GC pig depicting a fibrosis (blue) score of 4.9. (**F**) An example of the tissue response to CarboCell depots (*) with mononuclear cells and fibroblasts. Image G is a magnification of the highlighted area in image F. Scale bar = 200 μm (**D–F**) and 50 μm (**G**).

At 1 week, bone voids were filled with a fibrinous clot containing CarboCell, which in some cases was outlined by fibrous tissue that became more pronounced and organized at 3 weeks (Fig. 5A through C). Histopathology demonstrated that the bone void contained a mixture of fibrin, proteinous material, and CarboCell intermingled with necrotic bone debris and varying amounts of inflammatory cells, including mononuclear cells and neutrophils. The PBA was made up of an inner layer of granulation- and fibrous tissue, sometimes containing foci of hemosiderophages, mononuclear cells, and giant cells, with necrotic bone debris at the bone void border. Behind this, varying amounts of woven bone, with active osteoblasts, and osteoclasts were present. Foci of newly formed cartilage were present at 3 weeks in 9/10 pigs (Fig. 5D). The fibrosis score was higher in the CC2-GC compared to CC1-GC group (*P* < 0.05), and the osteoclast count was similar between groups (Table 1; Fig. 5E). Neutrophil counts were below the infection cutoff value for all pigs in the CC1-GC and CC2-GC groups, but above in all pigs in the CC1-G group (Table 1). All tissue sections were negative for *S. aureus* (Table 1), although other contaminating bacteria could be detected in two pigs in the CC1-G and one pig in the CC2-GC group. CarboCell was mainly present in the bone void and PBA but could also be seen outside the PBA in healthy bone tissue. The cellular response to CarboCell was generally mild, consisting of fibroblasts and mononuclear cells (Fig. 5F and G), but in some cases, mainly in the CC1-G group, there was a more pronounced inflammatory cell infiltration and necrosis in relation to some, but not all, CarboCell depots within the same tissue section. With the exception of unilateral pyelonephritis in a CC2-GC pig and kidney atrophy and unilateral cyst in a CC1-G pig (both common accidental findings in slaughter pigs), there were no macroscopic or histopathological kidney abnormalities.

**TABLE 1:** Bone histopathology in pigs treated with CC1-G, CC1-GC, or CC2-GC (mean ± SD or percentage)^*a*^

	CC1-G (*n* = 4)	CC1-GC (*n* = 5)	CC2-GC (*n* = 10)
*S. aureus* immunohistochemistry (% positive)	0	0	0
Neutrophil count (% above cutoff)	100	0	0
Osteoclast count (max. 100)	39 ± 14	20 ± 21	16 ± 13
Fibrosis score (max. 5)	3.8 ± 1.3^ab^	3.1 ± 1.2^a^	4.9 ± 0.3^b^

^*a*^ Values with different superscripts are significantly different (*P* < 0.05).

### Docusate and gentamicin are tolerated differently by osteoblasts *in vitro*

Docusate was tolerated by C2C12 osteoblast-differentiated cells at concentrations up to 10 µg/mL. Cell viability was not affected by any tested concentration of gentamicin (Fig. S2).

## DISCUSSION

This study demonstrates that IAO can be treated with revision surgery and local antibiotics alone, i.e., without systemic antibiotics. Specifically, local single- and dual-antibiotic treatment with gentamicin and clindamycin utilizing the CarboCell technology were effective in eradicating *S. aureus* in bone and soft tissue and prevented colonization of new implants in a clinically relevant pig model of IAO one-stage revision. Reformulation of gentamicin as a docusate ion-pair salt allowed co-formulation and sustained release of the intrinsically hydrophilic gentamicin and hydrophobic clindamycin from a single CarboCell formulation.

Previous preclinical studies have demonstrated that bacterial colonization and biofilm formation can recur with some drug delivery technologies (21, 30). As a testament to the CarboCell technology, neither a clinically approved hydroxyapatite–calcium sulfate bone void filler (21) or polymethylmethacrylate-based bone cement (30) containing gentamicin were able to eradicate *S. aureus* in one- or two-stage revision in large animal ODRI models, respectively, by local treatment alone. Additionally, it has been shown that debridement alone is unable to clear *S. aureus* infection in the porcine one-stage revision model (15). These results may, in part, relate to the extended high-dose release of CarboCell-formulated antibiotics observed in this study, which differs from the early short-lived release of antibiotics described for cement-based technologies (8). Moreover, the semi-solid nature of CarboCell allows for practically complete release of the incorporated antibiotics via a diffusion-dependent release mechanism. On the contrary, several cement-based technologies that are inherently solid have been demonstrated to release only a small fraction of the loaded antibiotic, even in long-term studies, which is also worrying from an AMR perspective (31). In this study, CarboCell release of gentamicin in bone tissue was 89±11% and 97±6 % after 1 and 3 weeks, respectively. In comparison, the release of gentamicin from cement-based technologies has been reported to be only 4%–12.5% after 1week (31–34).

In addition to antibiotic release profiles, several other factors could have contributed to the therapeutic efficacy of CarboCell. First, it may relate to chemical drug modification, as gentamicin–docusate HIP complexes encapsulated in different carriers have previously been shown to improve the antibacterial efficacy when compared to the unmodified free drug, albeit the underlying mechanisms are still largely unknown (10). Second, the ability to inject CarboCell into tissues and strategically distribute antibiotics throughout infected lesions in regions that may harbor bacteria is an important advantage compared to cement-based technologies that are restricted to bone voids. Lastly, co-formulation of antibiotics with differing capabilities in terms of spectrum, mode of action, and tissue- and cellular penetration may improve the therapeutic efficacy (3, 14, 35, 36). An additional advantage of combination therapy is that it may reduce the concentrations of antibiotics needed (3). To address this, we reduced the amount of administered CarboCell by approximately one-third and extended the treatment period to 3 weeks to determine prolonged effects. Despite the reduction in dose, the therapeutic efficacy was maintained at 3 weeks, with eradication of *S. aureus* in soft tissue, bone, and on implants, and early stages of bone healing were observed.

Surprisingly, an increased inflammatory tissue response was observed in the single- compared to dual-antibiotic groups. This could be associated with several factors, including a slower resolution of the infection and surgery-induced inflammation in the single-antibiotic group (24), drug-related reactions following differences in gentamicin and docusate release rates or altered stoichiometry of the released gentamicin–docusate HIP complexes between groups. In agreement with our cell viability data, a recent study has demonstrated that local concentrations of gentamicin up to 160,000 times the minimum inhibitory concentration for *S. aureus* were well-tolerated by porcine bone tissue (37). For docusate, we observed that *in vitro* cell viability was only affected above the critical micelle concentration (38, 39). Translation of this finding to an *in vivo* setting is complex, as docusate *in vivo* will be released into a milieu with high cell density and diversity and a vast membrane reservoir allowing for distribution and uptake of docusate. The increased complexity of tissue has been suggested to, in part, account for differences in *in vitro* and *in vivo* toxicity reported between HIP-modified and HIP-unmodified drugs, where HIP-modified drugs perform equal to or better than unmodified drugs *in vivo* in terms of safety (10). Notably, we observed that active osteoblasts were present in porcine bone tissue, also in close approximation to CarboCell depots. To better understand the translatability of our *in vitro* findings, measurements of local tissue concentrations of docusate and assessment of the viability of cells isolated from tissue are needed. In contrast to the single-antibiotic composition, both dual-antibiotic compositions displayed neutrophil infiltration below the infection threshold, and signs of active bone reorganization and healing could be observed after 3 weeks. Future studies should optimally include longer-term evaluation of tissue tolerability.

In conclusion, findings from this study highlight the robust therapeutic efficacy of both single- and dual CarboCell formulations against *S. aureus* IAO achieved without the need for systemic antibiotic therapy. This underscores the potential of precisely engineered antibiotic release technologies in addressing the difficulties associated with ODRI treatment, including high recurrence rates and the need for long-term systemic antibiotic therapy and AMR. Moving forward, continued research and development in this domain are warranted, with the ultimate aim of translating these advancements into tangible clinical benefits for patients with ODRI.

## Data Availability

Data are available upon request from the corresponding author.

## SUPPLEMENTAL MATERIAL

Supplemental material aac.00691-24-s0001.docx Fig. S1 and S2; Table S1.

ASM does not own the copyrights to Supplemental Material that may be linked to, or accessed through, an article. The authors have granted ASM a non-exclusive, world-wide license to publish the Supplemental Material files. Please contact the corresponding author directly for reuse.
