# Supplementary material for: Gentamicin and clindamycin antibiotic-eluting depot technology eradicates S. aureus in an implant-associated osteomyelitis pig model without systemic antibiotics
Source: Antimicrob Agents Chemother. 2024 Sep 17;68(10):e00691-24. doi: 10.1128/aac.00691-24 (PMC11459913; doi:10.1128/aac.00691-24)
Supplement: Supplemental material — Fig. S1 and S2; Table S1. [file aac.00691-24-s0001.docx]

Figure S1


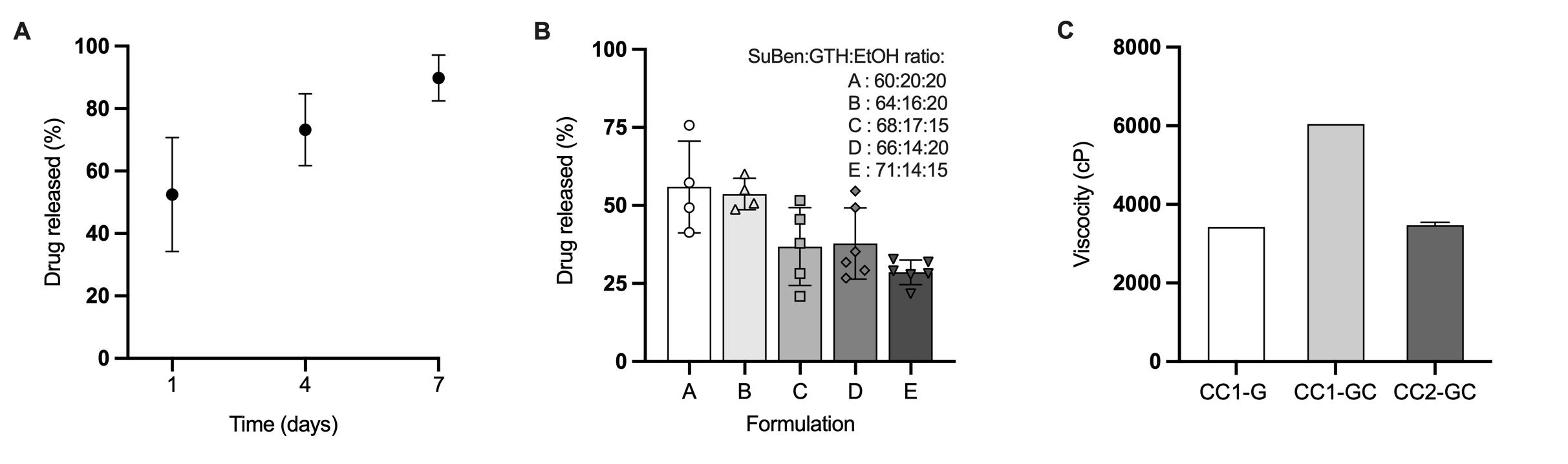


**Figure S1. Data foundation for optimization of CC1 and CC2 compositions**. **A** *In vivo* release of gentamicin-docusate from a SuBen:GTH:EtOH 60:20:20 composition as a function of time after a 50 $\mu$L subcutaneous injection of CarboCell in mice (n=6 at day 1 and 4, n=4 at day 7). The CarboCell formulation comprised 20mg/g gentamicin (74mg/g gentamicin-docusate). **B** *In vivo* release of gentamicin-docusate evaluated after 24 hours as a function of CarboCell composition after a 50 $\mu$L subcutaneous injection of CarboCell in mice (n=4-6). Composition A-D comprised 50 mg/g gentamicin (185 mg/g gentamicin-docusate) and composition E comprised 30mg/g gentamicin (111 mg/g gentamicin-docusate). All samples were analysed by HPLC and reported as mean$\pm$SD. **C** Viscosity of composition CC1-G, CC1-GC and CC2-GC at 25^o^C recorded using an EMS-1000 viscometer (spin 1000 rpms, 4.7 mm sphere).

Figure S2


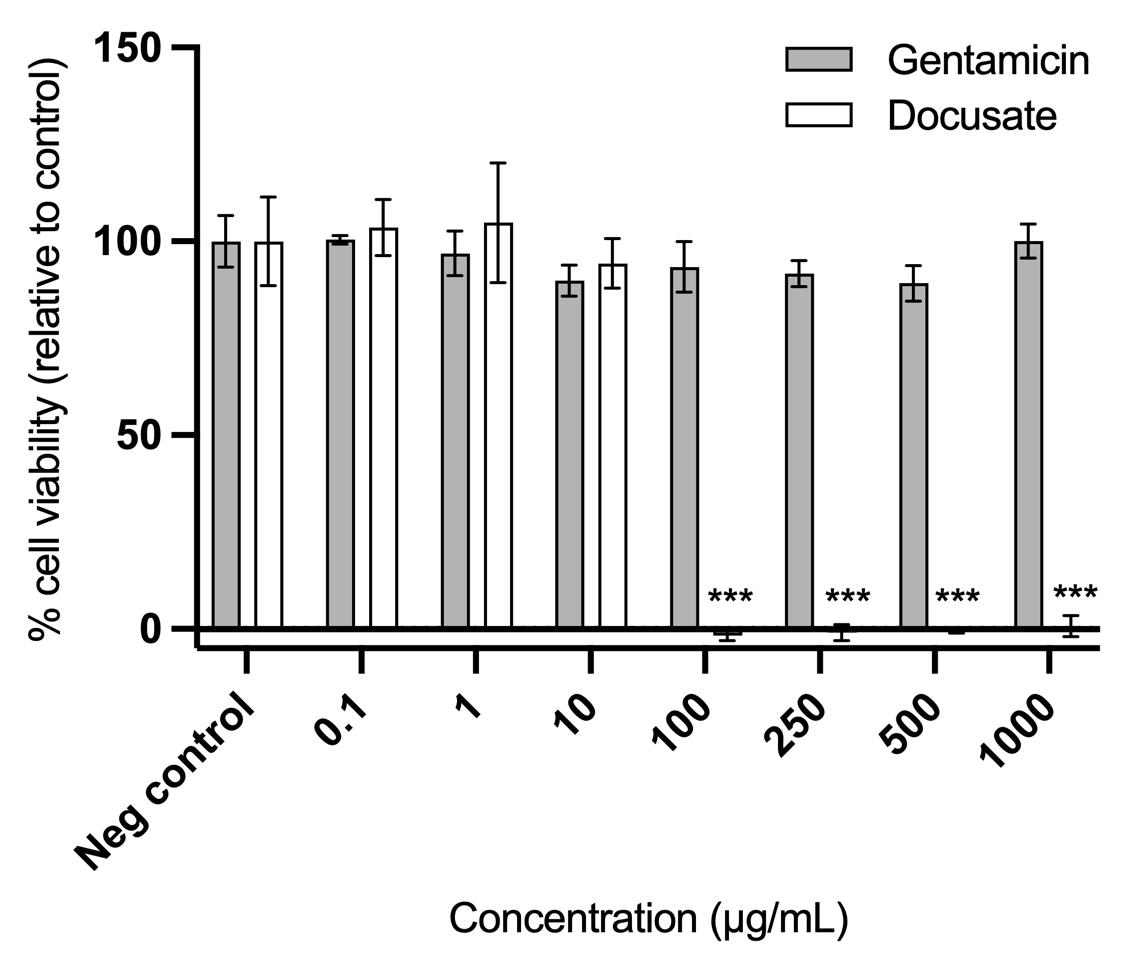


**Figure S2. Osteoblast cell viability.** Cell viability of C2C12 osteoblast-differentiated cells after exposure to different concentrations of gentamicin or docusate for 24 hours. Data are shown as percentage cell viability relative to the negative control. Triple asterisks denote a statistical probability level below 0.001.

Table S1

**Table S1**: Modified disk diffusion assay of surviving colonies in soft tissue of pigs treated with CC1-G or CC1-GC.

| **Sample** | | | **Clearing zone radius (mm)** | |
| --- | --- | --- | --- | --- |
| **Group/animal** | **Sample site** | **Colony size** | **Gentamicin** | **Clindamycin** |
| Wildtype - - | | | 9.3 | 15 |
| CC1-G/Pig 3 | Superficial | Large | 9 | - |
| CC1-G/Pig 4 | Superficial | Medium | 7 | - |
| CC1-G/Pig 4 | Superficial | Large | 9 | - |
| CC1-G/Pig 4 | Middle | Small | 9 | - |
| CC1-GC/Pig 5 | Superficial | Small | 10 | 15 |
| CC1-GC/Pig 5 | Superficial | Large | 10 | 14 |
| CC1-GC/Pig 5 | Middle | Small | 9 | 12 |
